# Is the College Too Young?

**Published:** 1920-11-27

**Authors:** 


### "IERNE" SEEKS INFORMATION.

### Is the College Too Young ?

A Strong Reason.

I had a strong reason for asking nurses to write to me. For some time it has been borne in upon me that they are not using the College as a means of achievement. Any lingering doubt that remained was swept away by Lord Knutsford at the recent "extraordinary" general meeting of the College. I once before observed that there is something very satisfactory about Lord Knutsford. There is no mistaking his drift. On this occasion he announced that the College is too young to achieve, and the statement would appear to have passed without contradiction or comment.

Why do not Nurses Vote?

I doubt if the twenty thousand women who comprise the College will agree with this attitude and policy of inactivity, and I want to find out if I am right. In addition to this, Sir Arthur Stanley, apparently in jest, referred to College electors consigning their voting papers to the waste-paper basket; and Lord Knutsford suggested that they used them as cigarette-lighters. The speakers would appear to think that this is a sign of complete confidence in the Council. Voting, to such happy members, is apparently a superfluity. I do not agree, but I may be wrong, and, if possible, this also I want to find out. There is an alternative explanation. There are people (like myself) who have always regarded College activity as feeble, and the Council as lacking in driving-power. This may be the result of an over-impulsive temperament, and such judgment may not be sound. I am neither defending nor condemning such a view, but merely pointing out that it exists.

My own limited knowledge and experience lead me to the belief that the more deeply interested men and women are in the clubs, institutions, or movements with which they have voluntarily associated themselves, the more active are they at elections of representatives, or where changes of policy and procedure are concerned. If the conditions are perfect, members are all the more nervous lest possibly some undesirable might find a place on the Council, and every precaution to prevent this is naturally taken.

**The failure of College members to vote is a danger signal, and it would be folly to underestimate its importance.**

How Long, O Lord, How Long?

Moreover, it must be obvious to any reflecting Councillor that an inactive non-voting member of the College is a worthless member. If there is to be, as there ought, an annual subscription, such a member may be a small financial asset, but so small as to be scarcely worth considering. The College ideal is progress, betterment—socially, mentally, financially. Members who take no interest in attaining to this ideal are of no value to the College or to the profession. I do not think that there are many such. But, as one who has watched the growth of the College since its inception, I should be surprised if there are not many who started out with enthusiasm and who have grown listless and apathetic. The College Council, it has always appeared to me, distinctly encouraged the phlegmatic temperament in its membership. Where reform was concerned it has ever been "Wait, wait, and keep on waiting"! Now, after three years of waiting, and when the nursing profession is in view of disaster, Lord Knutsford caps the climax by his airy "My dear, the College is quite too young to do anything."

If I may prophesy, unless this is promptly withdrawn, and convincing evidence produced to prove the contrary to be true, there will be much precious literature devoted to the lighting of cigarettes!

Pertinent or Impertinent.

Most pertinent to the issue is a question which I think demands a reply, but which I put with extreme reluctance, lest it may be regarded as impertinent. Lord Knutsford believes that nurses do not and cannot know for whom to vote. For some reason or other they elected him. Their motive I cannot hope to divine, but I think that he owes it to the public to explain what it was that prompted him to accept office. Why is he on the Council?

In his book, "Comrades in Captivity," F. W. Harvey describes some of the amenities of camp life in a German prison. Here is an extract:

"'What use are you to this camp?' This was the unasked question which had to be answered by every new arrival.

"It was hard rank Socialism, but very successful. You lived for the State, not for yourself.

"Could you write? There were camp papers needing contributors. Could you sing? There were concerts. Could you play games? The inter-barrack matches would give you the opportunity. Could you act? There was the little home-made theatre built to produce little home-made plays. Could you drink? There was the canteen, and those birthday evenings.

"Something you *must* do to keep the life of the camp alight. *Something* you must do, or be damned."

Some of the men in the prison were day and night on their knees scraping at a tunnel with scraps of wood, old nails, or their fingers, so that they and their comrades might find a way of escape. They were not too young, nor too short a time in prison. These men were in earnest.

IERNE.

